# Nasopharyngeal Carriage of *Streptococcus pneumoniae* in Healthy Children: Implications for the Use of Heptavalent Pnemococcal Conjugate Vaccine

**DOI:** 10.3201/eid0805.010235

**Published:** 2002-05

**Authors:** Paola Marchisio, Susanna Esposito, Gian Carlo Schito, Anna Marchese, Roberta Cavagna, Nicola Principi, the Hercules Project Collaborative Group

**Affiliations:** * University of Milan, Milan, Italy; † University of Genoa, Genoa, Italy

**Keywords:** *Streptococcus pneumoniae*, nasopharyngeal carriage, epidemiology, conjugate vaccine, children

## Abstract

We assessed the prevalence of *Streptococcus pneumoniae* serotypes in the nasopharynx of healthy children, antimicrobial susceptibility patterns, risk factors for carriage, and the coverage of heptavalent pneumococcal conjugate vaccine. In 2,799 healthy infants and children, the *S. pneumoniae* carrier rate was 8.6% (serotypes 3, 19F, 23F, 19A, 6B, and 14 were most common). Most pneumococci (69.4%) were resistant to one or more antimicrobial classes. The rate of penicillin resistance was low (9.1%); macrolide resistance was high (52.1%). Overall, 63.2% of the isolates belonged to strains covered by the heptavalent pneumococcal vaccine. This percentage was higher in children <2 years old (73.1%) and in those >2-5 years old(68.9%). Sinusitis in the previous 3 months was the only risk factor for carrier status; acute otitis media was the only risk factor for the carriage of penicillin-resistant *S. pneumoniae*. Most the isolated strains are covered by the heptavalent conjugate vaccine, especially in the first years of life, suggesting that its use could reduce the incidence of pneumococcal disease.

The nasopharynx of children has resident microbial flora that do not usually harm the child but, in some cases, constitute a reservoir of pathogens implicated in respiratory tract infections and invasive diseases (1,2). Because the bacteria carried in the nasopharynx of healthy children reflect the infection-causing strains currently circulating in the community (3), and so studies of the prevalence of different pathogens and their resistance patterns can provide useful indications for more rational therapeutic and preventive strategies.

The asymptomatic nasopharyngeal carriage of *Streptococcus pneumoniae* is widely prevalent in young children and has been related to the development of disease and the spread of the pathogen (4,5); furthermore, nasopharyngeal colonization by antibiotic-resistant S. pneumoniae has steadily increased over the last few years (6,7). Antibiotic-resistant strains are more often carried by infants and young children than adults and belong to a limited number of serotypes that are also some of the most common causes of invasive pediatric diseases (8–10).

A heptavalent conjugate vaccine, which includes the most common serotypes involved in invasive diseases, effectively induces protection against pneumococcal nasopharyngeal carriage (11,12). However, while the vaccine is statistically effective in preventing carriage of vaccine-related strains, a number of reports show an increase in the percentage of nonvaccine strains in immunized patients (13,14).

We assessed the prevalence of different *S. pneumoniae* serotypes in the nasopharynx of healthy children attending day-care centers and primary schools, determined their antimicrobial susceptibility to a wide range of therapeutic compounds, identified the risk factors for carrier status, and defined the possible coverage provided by the heptavalent pneumococcal conjugate vaccine during the first years of life.

## Patients and Methods

### Study Population

From April 15 through June 15, 2000, a single nasopharyngeal specimen per child was obtained from children attending day-care centers and the first years of primary schools in 13 Italian cities (3 northern, 4 central, 6 southern). Only one pediatrician, belonging to the main pediatric department in each city, was responsible for the study. Two day-care centers (one for infants and children <3 years of age and one for children ages 4-5 years) and one primary school (children ages 6-7 years) were also included. All of the children attending each participating center were considered eligible unless they had an underlying chronic illness (immunologic diseases; neoplastic disorders; renal, cardiac, hepatic, or hematologic diseases; bronchodysplasia; Down syndrome; chronic otitis media with effusion) or even a mild acute upper or lower respiratory tract infection at the time of enrollment.

The study protocol was approved by the ethics committees of the pediatric departments in charge of health control of the day-care centers and schools in each city, and written informed consent was obtained from a parent or guardian of each child. The questionnaires used to obtain demographic and clinical characteristics of the enrolled children were completed by trained reviewers in the presence of parents. The questions included: 1) the duration of breast-feeding; 2) living conditions (urban vs. rural); 3) information about previous day-care attendance; 4) the number and age of family members; 5) birth rank; 6) smoking habits of the family members living together; 7) the number and type of respiratory infections (including rhinitis, tonsillitis, laryngitis, acute otitis media, sinusitis, acute bronchitis, and pneumonia) during the previous 3 months; and 8) the number and type of antimicrobial drugs administered during the previous 3 months. The information was gathered without knowing the child’s carrier status.

### Specimen Collection

Nasopharyngeal specimens were obtained by the same trained investigator in each center on the basis of a previously described and validated protocol (15): a Mini-Culturette (Becton Dickinson, Cockeysville, MD) extra-thin flexible wire swab with its tip bent at an angle of approximately 30° was inserted through the mouth and placed 1 to 1.5 inches into the nasopharynx without touching the uvula or the tongue and kept in place for at least 5 seconds. The children were not allowed to eat or drink for 3 hours before specimen collection.

### Microbiologic Procedures

The same microbiologic procedures were used by all of the participating centers on the basis of previously validated guidelines (15,16). The nasopharyngeal cultures were injected into Stuart transport medium tubes (Venturi Transystem, Brescia, Italy), which were sent to the microbiology laboratory of the individual hospitals within 1-3 hours and immediately processed. S. pneumoniae was isolated and identified using standard laboratory procedures (16). The strains were stored in serum-glycerol freezing medium at -80°C, and the frozen samples were sent to the University of Genoa’s Institute of Microbiology to confirm their identity and to test their antimicrobial susceptibility by Gram strain morphology, catalase reaction, optochin susceptibility, and bile solubility. The strains were serotyped by their quellung reaction with sera produced by the Statens Seruminstitute (Copenhagen, Denmark) according to established procedures (16).

The strains susceptibility to penicillin, amoxicillin, amoxicillin-clavulanic acid, cefotaxime, ceftriaxone, meropenem, erythromycin, clarithromycin, azithromycin, tetracycline, trimethoprim-sulfamethoxazole, rifampicin, and chloramphenicol was tested using the agar dilution method described by the National Committee for Clinical Laboratory Standards (16). MICs of the antimicrobial drugs were determined using the Sensititre microbroth incorporation technique with an inoculum of approximately 105 CFU/mL in a medium of Mueller-Hinton broth supplemented with saponin-lysed horse blood and Factor V. Further details concerning the microbiologic method we used are given in the article describing the results of the Alexander Project (16). All of the laboratory work was carried out in a blinded manner; susceptibility was tested by a technician unaware of the serotypes, and the samples were typed by a technician unaware of the susceptibility results.

### Data Management and Analysis

The association between the characteristics of the children and the carriage of *S. pneumoniae* was first analyzed by a series of univariate analyses. Then, to control simultaneously for the possible confounding effects of the different variables, the risk for being an *S. pneumoniae* carrier was estimated by multiple logistic regression analysis with stepwise variable selection. The univariate and multivariate analyses of antibiotics and antibiotic resistance assessed individual drugs as well as all antibiotics together. In both analyses, the association was expressed in odds ratios (OR) and 95% confidence intervals (CI). Logistic regression analyses were made to evaluate the correlates of the carriage of different *S. pneumoniae* strains. On the basis of previous studies (12,17–19), strains 14, 6, 19, 18, 23, 9, 1, 7, 4, 5, 3, and 24 were considered invasive, and strains 4, 6, 9, 14, 18, 19, and 23 covered by or cross-reactive with the heptavalent vaccine. All of the reported pvalues are two-sided and refer to a significance level of 0.05.

## Results

### Study Participation

The study involved 2,799 children, whose demographic and clinical characteristics are shown in Table 1. Most of them lived in an urban area, attended a large day-care center full-time, belonged to small families, and had at least one respiratory tract infection in the previous 3 months.

**Table 1:** Characteristics of the 2,799 children tested for nasopharyngeal carriage of S*treptococcus pneumoniae*, 13 Italian cities, April 15–June15, 2000

Characteristics	No. of children
(% of 2,799)
Male	1,459 (52.1)
Race (white)	2,760 (98.6)
Age	
<2 yrs	420 (15.0)
2-5 yrs	1,389 (49.6)
>5 yrs	990 (35.4)
Breast-feeding ≥3 mo.	1,477 (52.8)
Urban residence	2,537 (90.6)
Full-time child-care attendance^a^	2,571 (91.9)
No. of subjects in each child-care center	
<20	927 (33.1)
20-29	1,600 (57.2)
>29	272 (9.7)
No. of siblings	
0	752 (26.9)
1	1,450 (51.8)
>2	597 (21.3)
First-born	1,366 (48.8)
Passive smoking	1,320 (47.2)
URTIs in the last 3 mo.^b^	
Rhinitis	1,759 (62.8)
Tonsillitis	636 (22.7)
Laryngitis	501 (17.9)
Acute otitis media	558 (19.9)
Acute sinusitis	174 (6.2)
LRTIs in the last 3 mo.^b^	
Acute bronchitis	579 (20.7)
Pneumonia	167 (5.9)
Antibiotic therapy in the last 3 mo.^c^	
Aminopenicillins	244 (8.7)
Amoxicillin-clavulanate	214 (7.6)
Macrolides	247 (8.8)
Cephalosporins	507 (18.1)
At least one antibiotic	1,032 (36.9)

^a^5-6 days/week, 6-8 hrs/day. ^b^One or more episodes; URTIs = upper respiratory tract infections; LRTIs = lower respiratory tract infections. ^c^ One or more courses.

### Recovery of *S. pneumoniae* from Nasopharyngeal Cultures

The pneumococcal carrier rate and the recovery of invasive strains or strains covered by the heptavalent vaccine are shown in Table 2. The total *S. pneumoniae* carrier rate was 242 (8.6%) out of 2,799, with no significant difference between the age groups; the most common strains were 3 (11.6%), 19F (11.2%), 23F (11.2%), 19A (10.7%), 6B (9.9%), and 14 (6.6%). Furthermore, 77.3% of the examined strains belonged to invasive serogroups (with no significant difference between the age groups) and 63.2% to serogroups covered by or cross-reactive with the heptavalent pneumococcal vaccine. The potential coverage related to the use of the heptavalent vaccine was significantly higher in children aged <2 (73.1%) or 2-5 years (68.9%) than in those aged >5 years (51.2%: <2 vs. >5 yrs: p=0.040; 2-5 vs. >5 yrs: p=0.0008). The proportion of invasive strains covered by the heptavalent vaccine was 153 (81.8%) of 187.

**Table 2:** Recovery of *Streptococcus pneumoniae* in the nasopharynx by age, 13 Italian cities, April 15–June 15, 2000^a^

	Age group (% of total/age group)			
Carriers	<2 yrs	2-5 yrs	>5 yrs	Total
Total/age group	420	1,389	990	2,799
Total carriers	26 (6.2)	132 (9.5)	84 (8.5)	242 (8.6)
Carriers of invasive strains	20 (74.1)	106 (81.5)	61 (71.2)	187 (77.3)
Carriers of strains covered by the heptavalent vaccine	19 (73.1)^a^	91 (68.9)^b^	43 (51.2)^ab^	153 (63.2)

^a^Carriers <2 yrs vs. carriers >5 yrs: p=0.040. ^b^Carriers 2-5 yrs vs. carriers >5 yrs: p=0.0008.

### Susceptibility Patterns

The antibiotic resistance pattern of the *S. pneumoniae* strains is shown in Table 3. Only 74 strains (30.6%) were susceptible to all of the antibiotics tested, 69 (28.5%) were resistant to one antimicrobial agent, 70 (28.9%) were resistant to two, and 29 (12.0%) to more than two.

**Table 3:** Antibiotic resistance pattern of *Streptococcus pneumoniae* strains, 13 Italian cities, April 15–June 15, 2000

Antibiotic	Resistant strains (%)
Penicillin	22 (9.1%)
Amoxicillin	0
Amoxicillin-clavulanate	0
Cefotaxime	9 (3.7)
Ceftriaxone	8 (3.3)
Meropenem	12 (4.9)
Azithromycin	126 (52.1)
Clarithromycin	126 (52.1)
Erythromycin	126 (52.1)
Tetracyclin	74 (30.6)
Thrimethoprim-sulphamethoxazole	58 (23.9)
Rifampicin	0
Chloramphenicol	26 (10.7)

Only 22 *S. pneumoniae* isolates (9.1%) were penicillin-resistant: 18 intermediately resistant (MIC 0.1-1.0 µg/L) and four fully resistant (MIC ≥2 µg/L); the serogroups most resistant were 9V (40.9%) and 23F (22.7%). Seventeen (77.3%) of these penicillin-resistant strains were preventable by the heptavalent pneumococcal conjugate vaccine; none of the five strains not covered by the vaccine is usually considered invasive. The incidence of penicillin resistance was significantly higher in younger children (25.9% in children aged <2 yrs vs. 7.6% in those aged 2-5 yrs: p=0.024; 25.9% in children aged <2 yrs vs. 5.9% in those aged >5 yrs: p=0.007).

Resistance to erythromycin, clarithromycin, and azithromycin (MIC_50_ 0.25, MIC_90_ ≥64) was very common (52.1%, 126 isolates); the most resistant serogroups were 6B (16.7%), 19F (15.9%), 14 (14.3%), and 19A (12.7%). Of these macrolide-resistant strains, 94 (74.6%) were preventable by the heptavalent pneumococcal conjugate vaccine; none of the 32 uncovered strains is usually considered invasive. Although no significant association was found, the macrolide-resistant strains were more often isolated in children aged <2 years (60.4%) than in those ages 2-5 years (56.3%) or >5 years (44.6%). Fourteen isolates (5.8%) were both penicillin and macrolide resistant.

### Risk Factors for Nasopharyngeal Carriage of *S. pneumoniae*

Table 4 shows the results of the univariate analysis of the potential risk factors for the nasopharyngeal carriage of *S. pneumoniae*. One or more episodes of sinusitis in the previous 3 months was the only risk factor for total carrier status and the carriage of both invasive strains and the strains covered by the heptavalent vaccine. None of the other variables was significantly associated with pneumococcal nasopharyngeal carriage, regardless of the strain. Multivariate analysis also indicated at least one episode of sinusitis in the previous 3 months as the only risk factor for the nasopharyngeal carriage of *S. pneumoniae* (total carriers: OR, 2.48; 95% CI 1.11-5.0; carriers of invasive strains: OR 3.04; 95% CI 1.23-6.53; carriers of strains covered by the heptavalent vaccine: OR 3.30; 95% CI 1.41-6.83).

**Table 4:** Univariate analysis of the variables potentially associated with the nasopharyngeal carriage of *Streptococcus pneumoniae*

	Total carriers		Carriers of invasive strains		Carriers of strains covered by the heptavalent vaccine	
Risk factor	ORs^a^	95% CI^b^	ORs	95% CI	ORs	95% CI
Sex	1.0	0.8-1.4	0.9	0.7-1.4	0.9	0.7-1.3
Age, yrs						
2-5 >5	0.7 1.5	0.1-1.7 0.9-2.5	1.3 0.9	0.8-2.3 0.5-1.6	1.5 1.1	0.9-2.7 0.6-1.9
Breast-feeding ≥3 mo.	0.9	0.6-1.4	1.0	0.6-1.7	1.1	0.7-1.7
Urban residence	0.8	0.1-2.2	1.2	0.6-2.6	1.5	0.7-2.9
Full-time day-care attendance	0.4	0.1-1.5	0.8	0.6-1.2	1.0	0.7-1.4
Child-care center ≥20 subjects	0.9	0.6-1.3	1.2	0.8-1.9	1.2	0.8-1.9
At least one sibling	0.9	0.7-1.3	0.7	0.4-1.2	0.7	0.4-1.3
First-born	1.0	0.8-1.4	1.1	0.8-1.6	1.1	0.8-1.6
Passive smoking	1.0	0.7-1.3	1.0	0.7-1.4	1.0	0.7-1.4
At least one URTIs^c^in the previous 3 mo.						
Rhinitis	0.9	0.7-1.3	1.0	0.7-1.5	1.0	0.7-1.5
Tonsillitis	0.8	0.6-1.3	0.9	0.6-1.5	1.0	0.6-1.5
Laryngitis	0.6	0.3-1.1	0.8	0.5-1.5	0.8	0.4-1.5
Acute otitis media	1.3	0.9-1.9	1.5	0.9-2.3	1.4	0.9-2.2
Acute sinusitis	2.3	1.1-4.6^d^	3.1	1.4-6.d	3.3	1.6-6.9 ^d^
LRTIs^e^ in the previous 3 mo.						
Acute bronchitis	0.7	0.4-1.1	0.6	0.3-1.1	0.6	0.4-1.1
Pneumonia	0.8	0.2-2.6	0.4	0.1-3.1	0.9	0.2-3.7
Antibiotic therapy in the previous 3 mo.						
Aminopenicillins	0.9	0.6-1.5	1.0	0.5-1.8	1.0	0.5-1.8
Amoxicillin-clavulanate	0.8	0.4-1.3	0.7	0.3-1.5	0.8	0.4-1.5
Macrolides	0.6	0.3-1.1	0.8	0.4-1.6	0.7	0.4-1.5
Cephalosporins	0.9	0.6-1.3	0.8	0.5-1.3	0.9	0.6-1.5
At least one antibiotic	0.8	0.7-1.2	1.0	0.7-1.4	1.0	0.7-1.4
^a^OR = odds ratio. ^b^ CI=95% confidence interval. ^c^ URTIs= upper respiratory tract infections. ^d^ p<0.05. ^e^ LRTIs=lower respiratory tract infections.						

In terms of antibiotic resistance, univariate analysis identified one or more episodes of acute otitis media in the previous 3 months as the only risk factor (OR, 2.8; 95% CI 1.2-6.8) and an age >5 years as a protective factor (OR, 0.3; 95% CI, 0.1-1.0) for the carriage of penicillin-resistant *S. pneumoniae*. Multivariate analysis confirmed the role of a previous history of acute otitis media (OR, 2.7; 95% CI, 1.0-6.6). Neither univariate or multivariate analysis identified an association between the carriage of macrolide-resistant strains and any risk or protective factor.

## Discussion

*S. pneumoniae* was carried by 8.6% of the 2,799 healthy children. Possible reasons for this low prevalence, similar to that previously reported by us (15), include the fact that the survey only included healthy children, the subjects were enrolled for a very short time, winter (a period of frequent respiratory illness) was not the season of enrollment, the large sample prevented any focus on specific situations, and the fact that human genetic traits may play a role (15,17,20). Furthermore, although a sampling or laboratory error is unlikely because all of the investigators were carefully pretrained and the microbiologic procedures were monitored throughout the study, some continuing colonization titers may have been below the sensitivity threshold of the culture method (13).

The serotypes most frequently colonizing our healthy population (3, 19F, 23F, 19A, 6B, and 14) were those commonly involved in invasive pneumococcal diseases (12,17–19,21), highlighting the importance of nasopharyngeal colonization in the development of serious community infections.

The low rate of penicillin resistance (9.1%) and the high rate of macrolide resistance (52.1%) detected in our study population are in contrast to the data reported in other countries (22–25) but consistent with previous Italian reports regarding adults and children with lower respiratory tract infections and invasive diseases (19,26,27). Comparison of the present data with those coming from our previous survey of a similar population of healthy subjects revealed an increased prevalence of antibiotic-resistant pneumococci, especially in children ages <2 years (15). As in other studies, we found that resistance to penicillin was associated with serotypes 9V and 23F, whereas resistance to macrolides was related to a wide range of serotypes (particularly 6B, 19F, 14, and 19A) (19,26,27).

Univariate and multivariate analyses indicate that infections of the nasal sinuses and the middle ear may favor *S. pneumoniae* carriage and may play a role in the spread of the organisms. However, considering that the data on the characteristics of our study population were obtained from parental recollection, the role of the different risk factors in pneumococcal colonization needs to be further confirmed. The protective effect of an age of >5 years on the carriage of penicillin-resistant strains is in agreement with other published data (17). As we have previously observed (15), but unlike other authors’ findings (10,28), nasopharyngeal carriage of S. pneumoniae and antibiotic use per se or the type of drug used in the previous 3 months were not related. In our previous survey (15), we found that having one or more older siblings and a history of full-time day-care attendance were risk factors for the nasopharyngeal carriage of *S. pneumoniae*, whereas living in a rural area was a protective factor. The differences observed in this study may be because of changes in the epidemiologic characteristics of pneumococcal carriage in Italy and confirm the importance of constant local surveillance.

Our data on the efficacy of the heptavalent pneumococcal conjugate vaccine indicate that it could have a considerable impact on the incidence of nasopharyngeal carriage and a major effect on invasive and antibiotic-resistant pneumococcal diseases, especially in the first years of life.

In conclusion, our study shows that, although nasopharyngeal carriage is low in healthy children, the most common circulating serotypes are invasive and antibiotic resistant. No risk factor other than sinusitis and acute otitis media seems to be related to pneumococcal colonization and to the carriage of penicillin-resistant *S. pneumoniae*, respectively*.* The fact that most isolated strains are covered by the heptavalent conjugate vaccine, especially in the first years of life, suggests that its broader use could reduce the incidence of pneumococcal disease.

## References

[1] Mackowiak PA. The microbial flora. N Engl J Med. 1982;307:83–93. 6806656 10.1056/NEJM198207083070203

[2] Faden H, Stniaevich J, Brodsky L, Bernstein J, Ogra PL. Changes in nasopharyngeal flora during otitis media of childhood. Pediatr Infect Dis J. 1990;9:623–6. 2122410

[3] Faden H, Duffy L, Wasielewski R, Wolf J, Krystofik D, Tung Y, Relationship between nasopharyngeal colonization and the development of otitis media in children. J Infect Dis. 1997;175:1440–5. 9180184 10.1086/516477

[4] Eldan M, Leibovitz E, Piglansky L, Raiz S, Press J, Yagupsky P, Predictive value of pneumococcal nasopharyngeal cultures for the assessment of nonresponsive acute otitis media in children. Pediatr Infect Dis J. 2000;19:298–303. 10.1097/00006454-200004000-00007 10783018

[5] Givon-Lavi N, Dagan R, Fraser D, Yagupsky P, Porat N. Marked differences in pneumococcal carriage and resistance patterns between day care centers located within a small area. Clin Infect Dis. 1999;29:1274–80. 10.1086/313465 10524975

[6] Zenni MK, Cheatham SH, Thompson JM, Reed GW, Batson AB, Palmer PS, *Streptococcus pneumoniae* colonization in the young child: association with otitis media and resistance to penicillin. J Pediatr. 1995;127:533–7. 10.1016/S0022-3476(95)70108-7 7562272

[7] Rudolph KM, Parkinson AJ, Reasonover AL, Bulkow LR, Parks DJ, Butler JC. Serotype distribution and antimicrobial resistance patterns of invasive isolates of *Streptococcus pneumoniae*: Alaska, 1991-1998. J Infect Dis. 2000;182:490–6. 10.1086/315716 10915080

[8] Kaplan SL, Mason EO, Barson WJ, Wald ER, Arditi M, Tan TQ, Three-year multicenter surveillance of systemic pneumococcal infections in children. Pediatrics. 1998;102:538–45. 10.1542/peds.102.3.538 9738174

[9] Block SL, Harrison CJ, Hedrick JA, Tyler RD, Smith RA, Keegan E, Penicillin-resistant *Streptococcus pneumoniae* in acute otitis media: risk factors, susceptibility patterns and antimicrobial management. Pediatr Infect Dis J. 1995;14:751–9. 8559623 10.1097/00006454-199509000-00005

[10] Deeks SL, Palacio R, Ruvinsky R, Kertesz DA, Hortal M, Rossi A, Risk factors and course of illness among children with invasive penicillin-resistant *Streptococcus pneumoniae.* Pediatrics. 1999;103:409–13. 10.1542/peds.103.2.409 9925833

[11] Black S, Shinefield H, Fireman B, Lewis E, Ray P, Hansen JR, Efficacy, safety and immunogenicity of heptavalent pneumococcal conjugate vaccine in children. Pediatr Infect Dis J. 2000;19:187–95. 10.1097/00006454-200003000-00003 10749457

[12] Eskola J, Kilpi T, Palmu A, Jokinen J, Haapakoski J, Herva E, Efficacy of a pneumococcal conjugate vaccine against acute otitis media. N Engl J Med. 2001;344:403–9. 10.1056/NEJM200102083440602 11172176

[13] Dagan R, Muallem M, Melamed R, Leroy O, Yagupsky P. Reduction of pneumococcal nasopharyngeal carriage in early infancy after immunization with tetravalent pneumococcal vaccines conjugated to either tetanus toxoid or diphtheria toxoid. Pediatr Infect Dis J. 1997;16:1060–4. 10.1097/00006454-199711000-00011 9384340

[14] Mbelle N, Huebner RE, Wasas AD, Kimura A, Chang I, Klugman KP. Immunogenicity and impact on nasopharyngeal carriage of a nonavalent pneumococcal conjugate vaccine. J Infect Dis. 1999;180:1171–6. 10.1086/315009 10479145

[15] Principi N, Marchisio P, Schito GC, Mannelli S. the Ascanius Project Collaborative Group. Risk factors for carriage of respiratory pathogens in the nasopharynx of healthy children. Pediatr Infect Dis J. 1999;18:517–23. 10.1097/00006454-199906000-00008 10391181

[16] Felmingham D, Gruneberg RN. the Alexander Project Group. A multicentre collaborative study of the antimicrobial susceptibility of community-acquired, lower respiratory tract pathogens 1992-1993: The Alexander Project. J Antimicrob Chemother. 1996;38(Suppl A):1–57. 10.1093/jac/38.4.747 8858472

[17] Sleeman K, Knox K, George R, Miller E, Waight P, Griffiths D, Invasive pneumococcal disease in England and Wales: vaccination implications. J Infect Dis. 2001;183:239–46. 10.1086/317924 11120930

[18] Hausdorff WP, Bryant J, Paradiso PR, Siber GR. Which pneumococcal serogroups cause the most invasive disease: implications for conjugate vaccine formulation and use, part I. Clin Infect Dis. 2000;30:100–21. 10.1086/313608 10619740

[19] Pantosti A, D’Ambrosio F, Tarasi A, Recchia S, Orefici G, Mastrantonio P. Antibiotic susceptibility and serotype distribution of *Streptococcus pneumoniae* causing meningitis in Italy, 1997-1999. Clin Infect Dis. 2000;31:1373–9. 10.1086/317502 11096005

[20] Gehanno P, Lenoir G, Barry B, Bons J, Boucot I, Berche P. Evaluation of nasopharyngeal cultures for bacteriologic assessment of acute otitis media in children. Pediatr Infect Dis J. 1996;15:329–32. 10.1097/00006454-199604000-00009 8866802

[21] Wee-Ling Soh S, Laa Poh C, Tzer Pin Lin RV. Serotype distribution and antimicrobial resistance of *Streptococcus pneumoniae* isolates from pediatric patients in Singapore. Antimicrob Agents Chemother. 2000;44:2193–6. 10.1128/AAC.44.8.2193-2196.2000 10898701 PMC90039

[22] Dagan R, Melamed R, Muallem M, Piglansky L, Yagupsky P. Nasopharyngeal colonization in Southern Israel with antibiotic-resistant pneumococci during the first 2 years of life: relation to serotypes likely to be included in pneumococcal conjugate vaccines. J Infect Dis. 1996;174:1352–5. 8940233 10.1093/infdis/174.6.1352

[23] Whitney CG, Farley MM, Hadler J, Harrison LH, Lexau CR, Reingold AL, Increasing prevalence in the United States of multidrug-resistant *Streptococcus pneumoniae.* N Engl J Med. 2000;343:1917–24. 10.1056/NEJM200012283432603 11136262

[24] Wenzel RP, Edmond MB. Managing antibiotic resistance. N Engl J Med. 2000;343:1961–3. 10.1056/NEJM200012283432610 11136269

[25] Tomasz A. New faces of an old pathogen: emergence and spread of multidrug-resistant *Streptococcus pneumoniae.* Am J Med. 1999;107(Suppl 1A):55S–62S. 10.1016/S0002-9343(99)00107-2 10451010

[26] Marchese A, Tonoli E, Debbia EA, Schito GC. Macrolide resistance mechanisms and expression of phenotypes among *Streptococcus pneumoniae* circulating in Italy. J Antimicrob Chemother. 1999;44:461–4. 10.1093/jac/44.4.461 10588306

[27] Principi N, Marchisio P. Epidemiology of *Streptococcus pneumoniae* in Italian children. Acta Paediatr. 2000;89:40–4. 10.1080/080352500300051530 11194797

[28] Levine OS, Farley M, Harrison LH, Lefkowitz L, Mc Geer A, Schwartz B. Risk factors for invasive pneumococcal disease in children: a population-based case-control study in North America. Pediatrics. 1999;103:1–5. 10.1542/peds.103.3.e28 10049984

